# The effects of alcohol use severity and polygenic risk on gray matter volumes in young adults

**DOI:** 10.3389/fpsyt.2025.1560053

**Published:** 2025-05-13

**Authors:** Yu Chen, Huey-Ting Li, Xingguang Luo, Guangfei Li, Jaime S. Ide, Chiang-Shan R. Li

**Affiliations:** ^1^ Department of Psychiatry, Yale University School of Medicine, New Haven, CT, United States; ^2^ Yale College, New Haven, CT, United States; ^3^ Department of Biomedical Engineering, College of Chemistry and Life Science, Beijing University of Technology, Beijing, China; ^4^ Beijing International Science and Technology Cooperation Base for Intelligent Physiological Measurement and Clinical Transformation, Beijing, China; ^5^ Department of Neuroscience, Yale University School of Medicine, New Haven, CT, United States; ^6^ Inter-Department Neuroscience Program, Yale University, New Haven, CT, United States; ^7^ Wu Tsai Institute, Yale University, New Haven, CT, United States

**Keywords:** HCP, alcohol dependence, polygenic risk score, VBM, thalamus

## Abstract

**Introduction:**

Genetic factors contribute to alcohol misuse. Chronic alcohol consumption is associated with decreases in gray matter volumes (GMVs) of the brain. However, it remains unclear whether or how genetic risks may alter GMVs independent of the effects of alcohol exposure.

**Methods:**

Here, we employed the Human Connectome Project data of neurotypical adults (n = 995; ages 22-35; 534 women) and, with voxel-based morphometry analysis, computed the GMVs of 166 regions in the automated anatomical atlas 3. Alcohol use behaviors were assessed with the Semi-Structured Assessment for the Genetics of Alcoholism. Alcohol use severity was quantified by the first principal component (PC1) identified of principal component analysis of 15 drinking measures. Polygenic risk scores (PRS) for alcohol dependence were computed for all subjects using the Psychiatric Genomics Consortium study of alcohol dependence as the base sample. With age, sex, race, and total intracranial volume as covariates, we evaluated the relationships of regional GMVs with PC1 and PRS together in a linear regression.

**Results:**

PC1 was negatively correlated with GMVs of right insula and Heschl’s gyrus, and PRS was positively correlated with GMVs of left posterior orbitofrontal cortex, bilateral intralaminar nuclei of the thalamus and lingual gyri.

**Discussion:**

These findings suggest distinct volumetric neural markers of drinking severity and genetic risks of alcohol misuse. Notably, in contrast to volumetric reduction, the genetic risks of dependent drinking may involve larger regional volumes in the reward, emotion, and saliency circuits.

## 1 Introduction

Genetic factors influence individual susceptibility to alcohol misuse, accounting for more than 50% of the variance in alcohol use severity, as demonstrated in twin and adoption studies (1–3). Polygenic risk scores (PRS), computed based on common genetic variants, predicted the severity of alcohol misuse (4, 5) and identified high-risk drinkers (6). However, the neural bases of the genetic risks of alcohol misuse remain largely unexplored. Characterizing the genetically informed neural phenotypes may help investigators distinguish the risks and consequences of misuse.

Chronic drinking is accompanied by widespread structural shrinkages of the frontal, parietal, temporal, and occipital lobes, the insula, as well as subcortical regions, including the amygdala, striatum, and thalamus (7–12). Drinking severity is correlated with lower regional gray matter volumes (GMVs) (13, 14) and functional loss in people with alcohol use disorder (AUD) (15). For instance, lower medial frontal and insular GMVs were associated with worse cognitive and emotional functions in AUD (16).

While this literature has described structural brain changes as the consequences of drinking, less research has aimed to identify brain markers of the genetic risks of alcohol misuse. An earlier study found larger bilateral insular surface area (SA) in AUD *vs*. healthy controls and identified 36 insular SA-related genes in significant correlation with AUD status with small effect sizes (*r* ^2^ < 0.000001) (17). Another study reported that alcohol-dependent individuals with two protective alleles of a single nucleotide polymorphism rs1789891 had reduced GMVs in bilateral superior, middle, and inferior temporal gyri (18). Luo and colleagues demonstrated significant and replicable association amongst KTN1 variants, KTN1 mRNA expression, and putamen GMVs in alcohol and comorbid substance misuse (19). Given that AUD is a complex, polygenetic disorder, investigating brain morphometric correlates of PRS for AUD can refine the prediction of individuals at risk for problem drinking.

Sex differences have been extensively investigated in the clinical manifestation as well as the neurobiology and pathophysiology of AUD (20). However, research aiming to identify sex differences in neural markers associated with genetic risks for alcohol dependence remains limited. Men relative to women have greater likelihood of early drinking, subsequent misuse, and higher lifetime prevalence of AUD (21). Imaging studies appeared to show mixed results regarding the effects of sex on neurochemical, structural, and functional mechanisms associated with AUD (22). A recent meta-analysis study revealed a group-by-sex interaction effect, with cerebellar GMVs more affected by AUD in women than in men and temporo-occipital and midcingulate GMVs more impacted in men as compared to women (23). An earlier study showed that male adolescents with *vs*. without family history of AUD had larger left hippocampal GMV, an effect not observed for females (24). Left nucleus accumbens GMV was positively correlated with family history of AUD in girls but not in boys (25). These findings indicate potential sex differences in genetic risks for alcohol misuse. Addressing sex differences in the neural markers of PRS is crucial to our understanding of the sex-specific vulnerability to the development of AUD.

In the current study, we aimed at identifying volumetric correlates of alcohol use severity and PRS for alcohol dependence and examining the sex differences in a cohort of young adults studied in the Human Connectome Project (HCP). We evaluated drinking severity, computed PRS for each subject, and correlated GMVs of 166 regions in the automated anatomical atlas 3 (AAL3) with drinking severity and PRS, with age, sex, race, and total intracranial volume (TIV) as covariates in the same model. We also performed the analyses separately for men and women and, in *post-hoc* comparisons, employed slope tests to confirm sex differences. We broadly hypothesized shared and unique volumetric correlates of alcohol use severity and PRS for alcohol dependence as well as significant sex differences in these correlates.

## 2 Methods

### 2.1 Dataset: subjects and assessments

In the S1200 release, the HCP contains clinical, behavioral, and 3T magnetic resonance imaging (MRI) data of 1,206 young adults (1,113 with structural scans) without severe neurodevelopmental, neuropsychiatric, or neurologic disorders. The current sample included 453 families with 1,140 healthy twins or siblings (ages 22-35; 618 women) with available genotyping data. A total of 145 subjects were excluded from further analyses due to missing drinking metrics (n = 65) or imaging data (n = 80). The final sample size was 995 (534 women).

Participants were assessed with the Semi-Structured Assessment for the Genetics of Alcoholism (SSAGA), an instrument designed to assess physical, psychological and social manifestations of alcoholism and related disorders (26). As in our previous study (27), we performed a principal component analysis on the 15 interrelated drinking metrics of the SSAGA in the HCP data, and identified the first principal component (PC1) with an eigenvalue > 1 that accounted for 49.74% of the variance. The PC1 value reflected drinking severity (“PC1” henceforth) and served as the phenotype in the PRS analysis.

### 2.2 MRI protocol and voxel-based morphometry

A customized 3T Siemens Connectome Skyra with a standard 32-channel Siemens receiver head coil and a body transmission coil was used in the MRI scanning. T1-weighted high-resolution structural images were acquired using a 3D MPRAGE sequence with 0.7 mm isotropic resolution (FOV = 224 × 224 mm, matrix = 320 × 320, 256 sagittal slices, TR = 2,400 ms, TE = 2.14 ms, TI = 1,000 ms, FA = 8°).

As described in our prior work (28, 29), we used voxel-based morphometry (VBM) to estimate the GMVs of brain regions with the CAT12 (Version 12.7) toolbox, following the suggested defaults (30): 1) individuals’ structural images were spatially normalized to the same stereotactic space; 2) the normalized images were segmented into gray matter, white matter, and cerebrospinal fluid; and 3) the gray matter (GM) images were smoothed. Specifically, a spatial adaptive non-local means denoising filter was first applied to the initial voxel-based processing (31), followed by internal resampling to accommodate low-resolution images and anisotropic spatial resolutions. Subsequently, data were processed by bias-correction and affine-registration, followed by the unified segmentation (32). After segmentation, the brain was parcellated into left and right hemisphere, subcortical areas, and cerebellum after skull-stripping. A local intensity transformation of all tissue classes was performed to reduce the effects of higher GM intensities in the motor cortex, basal ganglia, or occipital lobe, followed by adaptive maximum a posteriori segmentation with partial volume estimation (33). The tissue segments were then spatially normalized to a common reference space using DARTEL registrations (34). The GM maps were smoothed by convolution with an isotropic Gaussian kernel (FWHM = 8 mm). Data quality was checked by using the modules of display slices and VBM data homogeneity in the CAT12. The TIV estimated for each subject was used as a covariate in the following analyses.

### 2.3 Genotyping and PRS

The procedures of genotyping and PRS computation were described in detail in our prior work (35). Briefly, the discovery (base) sample included 14,904 unrelated cases with AUD (DSM-IV) and 37,944 unrelated healthy controls from the meta-analysis of ancestry-stratified genome-wide association (GWAS) of European and African-American cohorts by the Psychiatric Genomics Consortium (PGC) (36). Cases were not excluded based on the presence of Axis I or Axis II psychiatric disorders, while controls were defined as individuals with a lifetime history of alcohol consumption who did not meet criteria for alcohol abuse or dependence. No exclusions based on other psychiatric diagnoses were made because of their frequent comorbidity (36). The discovery sample was genotyped on microarrays and then imputed for 10,901,146 single nucleotide polymorphisms (SNPs). The current, HCP target sample was genotyped using Illumina Infinium Multi-Ethnic Genotyping Array (MEGA) or Infinium Neuro Consortium Array (2,292,654 SNPs). The computation of PRS requires base (i.e., discovery), target, phenotype, and covariate data (37). The summary statistics of GWAS served as the base data, and the whole-genome genotype and phenotype data (i.e., PC1) of the HCP sample served as the target data.

#### 2.3.1 Quality control for both base and target samples

We performed quality control on both base and target data based on a standard protocol (37, 38), to filter out the SNPs with ambiguous alleles or those mismatched between base and target data, and to exclude the subjects with sex mismatching between self-report and sex chromosomes. We also removed the samples with genetic relatedness for any pair of individuals between base and target samples. Finally, we checked the QQ plot of all *p* values from the cleaned base dataset and estimated the genomic inflation factor (λ), to further filter out the SNP outliers until the genomic inflation remained reasonably low. Only the SNPs present in both “cleaned” base and target samples were included for PRS computation. The final base data had a *λ* = 1.04 and chip-heritability > 0.05, and the risk alleles all had a *Z*-score > 1 for the effect size of SNP-AUD associations.

#### 2.3.2 PRS calculation

PRS was computed according to a formula introduced by Choi et al. (37) that considered the effect size of SNPs, the number of risk alleles, and the total number of SNPs included. In brief, PRS was computed as a sum of the genome-wide risk alleles, weighted by the corresponding effect size estimated from GWAS. We calculated the PRS for each subject using the program PRSice-2 (37), which automatically excluded the correlated SNPs (pairwise *r* ^2^ > 0.25) and considered age, sex (for all), and principal components of ancestry as covariates. The PRS for each subject were calculated using unlinked SNPs, with covariates including the first six ancestry principal components, age and sex, and the phenotypic variation explained by these PRSs was then determined (39).

To ensure that the subjects were unrelated within each target data subset, as required for the computation of the PRS, the twins and siblings of each family in HCP samples were randomly separated into independent target subsets. To maximize the sample size of each subset, the subjects within the first subset who were unrelated to the subjects of the other subsets were filled in each of the latter subsets repeatedly. The PRS and the percentage of phenotypic variation explained by the PRS (i.e., *R* ^2^) were then calculated separately for each subset under the same p-value thresholds. The PRS of the same subject, if showing a difference < 1% across the subsets, were averaged as the final PRS. The percentages of phenotypic variation, if showing a difference < 1% across the subsets, were averaged as the final percentages.

With age, sex (for all), and race as covariates, we computed the correlation coefficients between PC1 and PRS across all subjects as well as in men and women separately.

### 2.4 Region of interest analyses of GMV

We estimated the GMVs of 166 regions in the AAL3 (**Supplementary Table S1**) (40) and computed the correlation coefficients of regional GMVs with PC1 and PRS in a single regression model, controlling for age, sex (for all), race, and TIV in men and women combined and separately. For the correlations that were significant for either men or women alone, we also performed slope tests to confirm sex differences in the correlations (41, 42). Note that this analysis did not represent “double-dipping” as a correlate identified in men may have just missed the threshold in women and vice versa. Thus, slope tests were needed to confirm sex differences. We also conducted regression analyses on the volumetric correlates, with PC1, PRS, sex, and their interactions (PC1×sex and PRS×sex) as regressors, while controlling for age, race, and TIV.

In addition to the ROI analyses, we also performed whole-brain regressions against PC1 and PRS in a single model to identify the volumetric correlates, with the same covariates, in all and in men and women alone. We evaluated the results at voxel *p* < 0.001, uncorrected along with cluster *p* < 0.05 corrected for family-wise error (FWE) of multiple comparisons, based on the Gaussian random field theory, as implemented in the SPM12. However, we did not observe any significant clusters showing correlations with PC1 or with PRS in men and women combined or separately in the whole-brain analyses. Thus, we presented the results of ROI analyses on the 166 regions of AAL3 in the below.

## 3 Results

### 3.1 Demographic and clinical measures

The descriptive statistics and *t/χ²* tests of sex differences in age, race, PC1, PRS, and TIV are shown in **Table 1**. Women were older than men (*t* = 7.32, *p* < 0.001). There was no significant sex difference in race composition (*χ*² = 3.22, *p* = 0.666). With age and race as covariates, men *vs*. women showed significantly higher PC1, PRS, and TIV (*t’s* ≥ 11.07, *p’s* ≤ 0.015).

**Table 1:** Demographic and clinical measures of men and women.

Variables	Men (n = 461)	Women (n = 534)	*t/χ*²	*p*
Age (years)	27.86 ± 3.58	29.53 ± 3.61	-7.32	< 0.001
Race			3.22	0.666
* AI/AN*	1 (0.22%)	4 (0.75%)		
* A/NH/OPI*	25 (5.42%)	28 (5.24%)		
* AA*	55 (11.93%)	84 (15.73%)		
* White*	360 (78.09%)	396 (74.16%)		
* > one race*	12 (2.60%)	15 (2.81%)		
*UK/NR*	8 (1.74%)	11 (2.06%)		
Drinking PC1	0.39 ± 1.07	-0.34 ± 0.79	11.01	< 0.001
PRS	3.49 ± 0.80	3.38 ± 0.82	2.44	0.015
TIV (ccm)	1620.7 ± 157.2	1392.2 ± 125.0	23.03	< 0.001

Values are mean ± SD or n (%). AI/AN, American Indian/Alaskan Native; A/NH/OPI, Asian/Native Hawaiian/Other Pacific Islander; AA, African American; UK/NR, Unknown/Not Reported; PC1, first principal component of drinking metrics; PRS, polygenic risk score. A *χ*² test was used to examine sex differences in race. Independent *t* tests for PC1, PRS, and TIV were performed with age and race as covariates.

With age, sex (for all), and race as covariates, PC1 and PRS were significantly correlated in all subjects (*r* = 0.152, *p* = 0.000002) as well as in men (*r* = 0.130, *p* = 0.005235) and women (*r* = 0.174, *p* = 0.000055) separately. The slope test showed that the sex difference in the correlations was not significant (*t* = 0.114, *p* = 0.908915).

### 3.2 Volumetric correlates of PC1 and PRS

**Figure 1:**
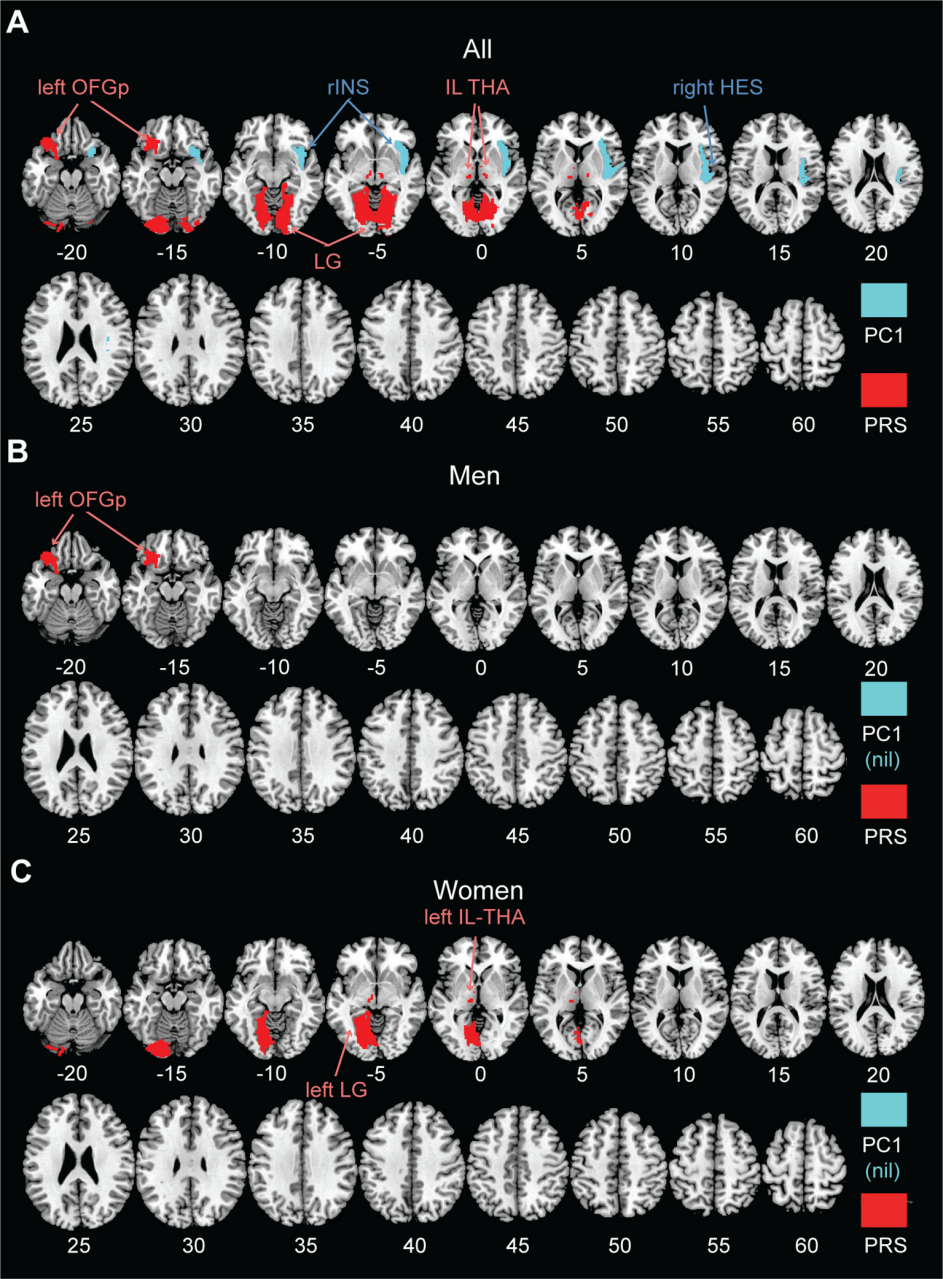
AAL3 regional GMVs in significant correlation with PC1 (cyan; negative) and PRS (red; positive) in a single regression model for **(A)** all, **(B)** men, and **(C)** women, with age, sex (for all), race, and TIV as covariates. No regions showed positive correlations with PC1 or negative correlation with PRS at the same threshold. PC1, first principal component of drinking metrics; PRS, polygenic risk score; OFGp, posterior orbitofrontal gyrus; INS, insula; IL-THA, intralaminar nucleus of thalamus; HES, Heschl’s gyrus; LG, lingual gyrus; nil, no significant findings.

**Figure 1** shows the volumetric correlates of PC1 and PRS in all, men, and women, with PC1 and PRS modeled together and age, sex (for all), race, and TIV as covariates. Specifically, for all subjects, higher PC1 was correlated with lower GMVs of the right insula (INS; *r* = -0.126, *p* = 0.000068) and Heschl’s gyrus (HES; *r* = -0.126, *p* = 0.000074). Higher PRS was correlated with higher GMVs of left posterior orbitofrontal gyrus (OFGp; *r* = 0.128, *p* = 0.000053), and bilateral intralaminar nuclei of the thalamus (IL-THA; left: *r* = 0.147, *p* = 0.000003 and right: *r* = 0.144, *p* = 0.000005) and lingual gyri (LG; left: *r* = 0.132, *p* = 0.000030 and right: *r* = 0.125, *p* = 0.000077). No regions showed positive correlations with PC1 or negative correlation with PRS. **Table 2** summarizes these volumetric correlates for both PC1 and PRS as well as the slope test results. With a *p*-value of 0.007 (0.05/7) to correct for multiple comparisons on slope tests, all volumetric correlates were unique to either PC1 or PRS, except for the left lingual gyrus.

**Table 2:** Correlations of volumetric correlates with PC1 and PRS and slope tests.

GMV	PC1	Effect	PRS	Effect	Slope test	
	*r*	Size *r^2^*	*r*	Size *r^2^*	*t*	*p*
All						
right INS	**-0.126**	0.01588	0.027	0.00073	3.599	3.2733e-04*
right HES	**-0.126**	0.01588	0.007	0.00005	3.181	0.0015*
left OFGp	-0.016	0.00026	**0.128**	0.01638	2.985	0.0029*
left IL-THA	-0.094	0.00884	**0.147**	0.02161	5.276	1.4612e-07*
right IL-THA	-0.056	0.00314	**0.144**	0.02074	4.289	1.8772e-05*
left LG	0.015	0.00023	**0.132**	0.01742	2.325	0.0202
right LG	-0.019	0.00036	**0.125**	0.01563	2.992	0.0028*
Men						
left OFGp	-0.011	0.00012	**0.200**	0.04000	2.691	0.0073
Women						
left IL-THA	-0.033	0.00109	**0.185**	0.03423	3.577	3.6295e-04*
left LG	-0.005	0.00003	**0.166**	0.02756	2.816	0.0050*

GMV, gray matter volumes; PC1, first principal component of drinking metrics; PRS, polygenic risk score; OFGp, posterior orbitofrontal gyrus; INS, insula; IL-THA, intralaminar nucleus of thalamus; HES, Heschl’s gyrus; LG, lingual gyrus. Bold *r* values represent significant correlations at corrected *p* < 0.05/(166×2) = 0.00015. *p < 0.05/7 = 0.00714 for multiple corrections on slope tests.

For men or women considered separately, PC1 was not significantly correlated with the GMVs of any AAL3 regions. In men, higher PRS was associated with higher GMV in the left OFGp (*r* = 0.200, *p* = 0.000016). In women, higher PRS was associated with higher GMVs in the left IL-THA (*r* = 0.185, *p* = 0.000019) and LG (*r* = 0.166, *p* = 0.000120). Moreover, slope tests confirmed that, in women, the correlations of PRS with left IL-THA GMV (*t* = 3.577, *p* = 3.6295e-04) and with left LG GMV (*t* = 2.816, *p* = 0.0050) were stronger than those with PC1. The correlation of the left OFGp GMV with PRS was also stronger than that with PC1 in men, although the *p* value (0.0073) just missed the corrected threshold. However, slope tests did not show significant sex differences in any of these GMV correlations with PRS (*t*’s ≤ 1.421, *p*’s ≥ 0.0729).

The linear regression models showed that the PRS×sex interaction was significant for the left OFGp only (*β* = 0.157, uncorrected *p* = 0.014), indicating a stronger correlation between PRS and left OFGp GMV in men (*r* = 0.200) than in women (*r* = 0.056). The PC1×sex interaction was not significant for any volumetric correlates (*p*’s ≥ 0.146).

## 4 Discussion

We aimed to distinguish regional volumetric markers of the genetic risks, as indexed by the PRS, for alcohol misuse from those reflecting the effects of alcohol exposure, as indexed by drinking severity PC1. To this end we employed a single regression model where both PRS and PC1 served as independent variables or predictors, so that the effects of genetic risks and alcohol exposure were mutually accounted for. We observed distinct volumetric correlates of PRS and PC1 and discussed the main findings in the below.

### 4.1 Volumetric correlates of drinking severity

We identified gray matter atrophy in the right insula and Heschl’s gyrus in relation to drinking severity, in line with prior reports (43). Insular GMV loss has been observed in drinkers (44), smokers (28), and other drug users (45) who often engage in heavy alcohol use. A critical hub of the interoceptive system, the insula is affected by alcohol’s neurotoxicity both structurally and functionally, and insula dysfunction contributes to the development of addiction (46). The current finding of insular GMV loss in a sample of largely non-dependent drinkers suggested that volumetric reduction of the insula can transpire early in the course of alcohol use.

Notably, a recent study with the HCP data demonstrated significant negative correlations between alcohol consumption and GMVs of right insula and middle/superior frontal gyri and attributed these volumetric alterations to shared genetic factors, based on the correlations of monozygotic *vs*. dizygotic twins (47). In the latter study, a concordant sibling pair was defined as a pair who were both in the same category of consumption (i.e., high or low). Conversely, a pair was considered discordant if the siblings belonged to different consumption categories. Siblings in the concordant high and low group each had lowest and highest insula and frontal GMVs, respectively. On the other hand, the GMVs did not differ between low and high alcohol-using siblings within the discordant pairs. The findings suggested genetic risks rather than alcohol use as the primary driver of GMV reductions. In contrast to our study, however, the latter study did not directly control for the influence of alcohol use when investigating the relationship between genetic risks and insular GMV. Alcohol exposure may play a role in shaping the association. Insular GMV as a genetic risk marker of alcohol misuse needs to be revisited in future studies, including those of patients with AUD.

The Heschl’s gyrus supports auditory and semantic processing (48, 49) but its role in substance misuse is less clear. Consistent with our findings, lower GMV in the left Heschl’s gyrus was associated with higher drinking severity in young and middle-aged adults (13). A few meta-analyses also showed GMV deficits in Heschl’s gyrus in patients with other substance misuse, including opioid (50) and cocaine (51) dependence and misuse of multiple substances (52), where heavy drinking has frequently been observed.

### 4.2 Volumetric correlates of the genetic risks of AUD

We computed PRS using the GWAS data for AUD and found that the PRS was significantly associated with drinking severity in our sample of young adults. With drinking severity accounted for, we found PRS in positive correlation with regional GMVs in left posterior orbitofrontal gyrus, bilateral intralaminar nuclei of the thalamus, and lingual gyri. These volumetric correlates may represent a genetically informed risk marker of alcohol misuse. A recent Adolescent Brain Cognition Development study showed that higher PRS for problem alcohol use was associated with greater cortical thickness of the right supramarginal gyrus in substance-naïve children (53). Substance-naïve offsprings of AUD patients have been found to have thicker frontal and temporo-parietal cortices (54), and many of these cortical regions are thinner in individuals with alcohol dependence (14). Although GMV and cortical thickness are different neural metrics, these previous reports are consistent with the current findings of distinct volumetric markers of PRS and severity of alcohol misuse.

It has been postulated that greater cortical thickness among those at familial risk for alcohol problems reflects a developmental delay in neural pruning, placing children and adolescents at risk for substance and alcohol use, which, in turn, accelerates neuronal pruning in later development. While no other studies to our knowledge have specifically reported larger regional volumes in link with the generic vulnerability of alcohol misuse, investigators have observed similar findings in clinical conditions comorbid with AUD. For instance, greater PRS for depression was associated with larger GMVs in bilateral hippocampi and right gyrus rectus (35) and right inferior and middle temporal gyri (55). It remains to be seen whether the comorbid conditions may share genetic bases.

Structural changes of the thalamus have long been documented in problem drinkers (7, 56, 57). In a study of meta-analysis, patients with AUD demonstrated significant GMV reduction in the left thalamus as compared to non-drinking controls, with reduction correlated with life-time alcohol consumption (7). No prior research has addressed whether thalamic subnuclei may be differentially impacted by alcohol or genetic risks for alcohol misuse. Here, we observed that bilateral intralaminar nuclei of the thalamus (IL-THA) GMVs were positively correlated with PRS, in contrast to volumetric reduction as observed for the thalamus as a result of chronic alcohol use. The IL-THA projects to cortical regions, dorsal and ventral striatum, and the amygdala, and supports physiological arousal, vigilance, and motivated behaviors (58). If replicated, these findings suggest that specific thalamic subnuclei, such as the IL-THA, may play a critical role in the neurobiological mechanisms underlying genetic risks for alcohol misuse. Studies combining genetic analyses and brain imaging can assess how intralaminar thalamic dysfunction may support ill-directed arousal to reward and/or negative emotions, disposing individuals to earlier and heavier drinking.

The orbitofrontal gyrus (OFG) supports goal-directed behaviors particularly through evaluation of rewards and other outcomes, which have been shown to be disrupted in substance misuse, including AUD (59). Within the OFG, the posterior region is primarily involved in emotion perception and processing (60, 61). Chronic alcohol use was associated with reduced OFG volumes (62). In addition, OFG function appears to be influenced by genetic variations in neurotransmitter receptors, such as the A-G allele variants in the OPRM1 receptor and the 7-repeat allele of the DRD4 receptor. For example, the OFG shows higher response to alcohol relative to neutral cues in heavy drinkers, with stronger activation observed in those carrying the OPRM1G allele, suggesting a genetic predispositions to alcohol dependence (63, 64). Other studies reported greater neuroinflammation in the OFG (65), which may contribute to changes in the OFG volumes (55, 66), in individuals with AUD. Although it is premature to attempt to reconcile these findings, the studies together suggest the importance of distinguishing genetic risks in the investigation of the pathophysiology of AUD.

The lingual gyrus, located in the occipital lobe and involved in visual processing (67), has been implicated in alcohol misuse in prior research. For instance, heightened activation in the bilateral lingual gyri has been associated with alcohol cue-induced craving among individuals with AUD (68). Interestingly, enlargement of visual processing regions, including the lingual gyrus, has been observed in anxiety and depressive disorders, which are common comorbidities of AUD (69, 70). Additionally, we recently demonstrated stronger resting-state functional connectivity between the subgenual anterior cingulate cortex and the lingual gyrus in association with higher PRS for depression (71). Future research should explore both structural and functional characteristics of the lingual gyrus in relation to genetic predisposition for alcohol dependence.

Taken together, the divergent patterns of association – greater GMVs in relation to PRS and reduced GMVs associated with PC1 – may reflect distinct underlying mechanisms. The “genetic risk-related increases” in GMV, particularly in the thalamus, OFG, and lingual gyrus, may represent neurodevelopmental deficits that precede alcohol use. These volumetric changes could indicate delayed synaptic pruning, heightened neural sensitivity to rewards, or altered arousal regulation that predispose individuals to initiation and/or escalation of alcohol use. In contrast, the “alcohol exposure-related atrophy” observed in the insula and Heschl’s gyrus likely reflects the neurotoxic effects of alcohol on brain structure, as mediated by excitotoxicity, oxidative stress, or neuroinflammation. The insula, in particular, is highly vulnerable to these substance-induced effects due to its dense interconnections and role in interoception and craving (72).

These findings suggest that brain structure may be shaped both by pre-existing genetic vulnerability and by the consequences of substance use, each affecting different regions and neural systems. This dual-pathway model underscores the importance of studying genetic predispositions and environmental exposures in tandem. Longitudinal and developmental studies will be essential to disentangling these effects and identifying when and how genetic risk manifests structurally, and how alcohol use may exacerbate or modulate these trajectories.

### 4.3 Potential sex differences?

The slope tests did not identify any significant sex difference in PRS-PC1 correlation or in the volumetric correlates of PC1 or PRS, despite the finding of both higher PRS and PC1 in men *vs*. women. In contrast, the linear regression models with interaction terms suggested a potential sex difference in the left posterior OFG GMV, although this finding did not survive correction for multiple comparisons. Earlier family, adoption, and twin studies found similar heritability estimates of alcohol dependence (73) and GMVs (74) across sexes. However, the patterns of morphological brain deficits as a result of chronic drinking appeared to differ between men and women (23, 75–78). Moreover, in alcohol-naïve adolescents with a family history of AUD, males exhibited larger left hippocampal GMV, a pattern that was not observed in their female counterparts (24). In another study, healthy female but not male adolescents with a family history of AUD showed larger GMV of the left nucleus accumbens (25). These findings suggest potential sex differences in the brain structure related to the genetic predisposition to alcohol misuse, as has been demonstrated for sex differences in brain functioning, e.g., the roles of dopaminergic signaling in risk/novelty seeking (79) and hormonal modulation of dopaminergic signaling (20), that may dispose individuals to early and more severe drinking. Further, GMV correlates of clinical conditions comorbid with problem drinking, including ADHD (80, 81) and depression (35) or of psychological constructs that conduce to problem drinking, including impulsivity (74, 82), manifest robust sex differences. Studies of larger sample sizes and with individuals with more severe drinking problems are needed to better understand how sex differences are reflected in the risk markers or inter-related outcomes of chronic alcohol consumption.

### 4.4 Limitations and conclusions

There are several limitations to consider. First, HCP participants were largely healthy (“neurotypical”) young adults without severe drinking problems. Thus, the current findings should be regarded as specific to this sample. In particular, no ROIs showed GMVs in negative correlation with PC1 in men or in women alone, a finding that likely reflects this young adult, largely social-drinking population. Future research involving clinical cohorts is needed to determine whether similar neural correlates of genetic and behavioral risks for AUD are observed in individuals with more severe or chronic patterns of alcohol use. Second, we did not consider other substance uses in our analyses. Nicotine or other drug use may have additive or interactive effects on regional GMVs (9, 83). Third, longitudinal studies are needed to better understand the distinct impacts of genetic risks and alcohol use on the brain.

In conclusion, we replicated GMV reduction in association with greater severity of alcohol use and provided evidence for regional GMVs in positive association with the genetic risks of alcohol dependence in young healthy adults. Our findings suggest potentially unique volumetric markers for drinking severity and genetic risks for alcohol dependence.

## Data Availability

Publicly available datasets were analyzed in this study. This data can be found here: Human Connectome Project (HCP) https://www.humanconnectome.org/study/hcp-young-adult.
